# Differential Diagnosis between Sintilimab-related Autoimmune Myocarditis and Acute Myocardial Infarction

**DOI:** 10.1186/s12575-025-00267-4

**Published:** 2026-03-03

**Authors:** Yihe Wu, Jiayun Nian, Hongxu Liu, Xiaolei Lai, Zihao Liu, Tengfei Li, Shenglei Qiu

**Affiliations:** 1 Beijing Hospital of Traditional Chinese Medicine, Capital Medical University, Beijing, China; 2 Beijing University of Chinese Medicine, Beijing, China

**Keywords:** Sintilimab, Immune checkpoint inhibitor, Autoimmune myocarditis, Acute myocardial infarction, Differential diagnosis

## Abstract

**Objective:**

To analyze the regularities and clinical features of sintilimab-related autoimmune myocarditis, and to summarize the differential diagnosis key points between sintilimab-related autoimmune myocarditis and acute myocardial infarction.

**Methods:**

The case reports about sintilimab-related autoimmune myocarditis were searched on databases from the establishment of the database to April 1st 2024. The relevant medical records were searched on the hospital information system of Beijing Hospital of Traditional Chinese Medicine in the past 3 years. The case reports and medical records were collected for statistical analysis.

**Result:**

Twenty three cases were collected including 22 case reports and 1 case record. Most of the sintilimab-related autoimmune myocarditis were in elderly men aged 60–75 years old and occurred between the end of the first dose of treatment to the beginning of the second dose. The symptom was nonspecific such as chest tightness and palpitation, sometimes with symptom of myasthenia as muscle weakness or myositisand as muscle soreness. Elevated cardiac biomarkers and changes in electrocardiogram were common, and decreased left ventricular ejection fraction was rarely seen in echocardiography. 9 cases underwent coronary angiography or computed coronary tomography angiography, and 3 cases underwent cardiovascular magnetic resonance.

**Conclusion:**

The manifestations of sintilimab-related autoimmune myocarditis are not specific. The medication history and concomitant symptoms are of warning value. Coronary angiography or coronary computed coronary tomography angiography can be helpful when ruling out acute myocardial infarction. Cardiovascular magnetic resonance and myocardial biopsy can confirm the diagnosis. Cardiac biomarkers and the electrocardiogram can assist in diagnosis and prognosis assessment.

## Introduction

Cardiovascular diseases and malignant tumors are two of the major diseases threatening human life and health, with their high morbidity and mortality [1]. The innovative development of cancer therapies has led to an unprecedented improvement in survival outcomes, and cardiovascular disease has become the second leading cause of mortality in cancer survivors after recurrent malignancy [2]. The development of onco-cardiology is in the ascendant, and cardiovascular adverse reactions caused by antineoplastic drugs are the research focus in this discipline.

Sintilimab (trade name: Darbosux) is a kind of Immune Checkpoint inhibitors (ICIs) which enhances T-cell responses in recognizing the specific tumor antigens and killing tumor cells, and thereby restore the antitumour T-cell response, and consequently plays an inhibitory role in tumor growth and immune escape [3, 4]. Sintilimab has been approved in China for the treatment of Hodgkin's lymphoma and a variety of solid tumors, including non-small-cell lung cancer, hepatocellular carcinoma and esophageal cancer, which has brought hope to many tumor patients [5–10]. However, adverse reactions introduced by the immunotoxicity of sintilimab have emerged during the treatment [11–13].Wherein autoautoimmune myocarditis characterized by acuteness, rapid transmission and high mortality after ICI therapy attracts increasing concern [14–17]. And the rapidly rising number of related case reports suggests that the incidence of autoimmune myocarditis may be underestimated [18]. Therefore, the identification and differential diagnosis of sintilimab-related autoimmune myocarditis has become a clinical challenge.

Sintilimab is a fully human IgG4 monoclonal antibody that binds to programmed cell death receptor-1 (PD-1), thereby blocking the interaction of PD-1 with its ligands (PD-L1) and consequently helping to restore the endogenous anti-tumour T-cell response [19]. However, the autopsy results of patients with myocarditis after using PD-1 inhibitor shows that the same T-cell recognition antigens are present in the infiltrating T cells of both myocardium and tumors. And this finding indicated that the T cells activated by sintilimab recognize not only the antigens on tumor cells, but also the high-isogeneic antigens on cardiomyocytes, resulting in autoimmune myocarditis [20–22]. In addition, PD-1/PD-L1 plays an important role in cardiac protection. PD-L1 can restrict the activation of specific-cardiac antigen cytotoxic T cells (CTL), thereby inhibiting their killing effect on cardiomyocytes. After the treatment of PD-1/PDL-1 inhibitors, the balance of cardiac peripheral immune tolerance is broken, and unrestricted CTLS are heavily activated and attack the heart, exacerbating the cardiomyocytes damage [23–25]. Meanwhile, inhibitions of PD-1 cause Treg cells turning into pro-inflammatory state, and the activity of CD4/CD8 increases. Activated immune cells release a large amount of inflammatory cytokines such as TNF-α, IL-17 and IL-6 into the circulatory system, leading to further damage of myocardial tissue [22].

Acute Myocardial Infarction (AMI) is a group of clinical syndromes caused by acute myocardial ischemia, usually presenting with acute episodes of chest pain, chest tightness, palpitations, fatigue and other symptoms, accompanied by elevated cardiac biomarkers and a series of characteristic electrocardiographic changes of myocardial ischemia or necrosis [26]. Appearing similar to AMI in clinical symptoms, elevated serum indexes, electrocardiogram (ECG) changes, and other aspects, sintilimab-related autoimmune myocarditis is easily misdiagnosed as AMI in clinical practice, thus missing the ptimal treatment stage [27]. Moreover, the therapeutic principles of autoimmune myocarditis and AMI contradict each other. The former is mainly treated with glucocorticoid for anti-inflammatory therapy [28], while glucocorticoid may increase the risk of complications such as heart rupture in AMI [29]. Therefore, rapid identification of sintilimab-related autoimmune myocarditis and accurate differentiation of AMI is of significant clinical importance.

In this work, case reports of sintilimab-related autoimmune myocarditis were collected academic resource retrieval websites and relevant medical records were collected from the hospital information system of Beijing Hospital of Traditional Chinese Medicine, Capital Medical University. The clinical features of sintilimab-related autoimmune myocarditis were extracted, and the key points of differential diagnosis of sintilimab-related autoimmune myocarditis and AMI were summarized to offer guidance for clinical recognition.

## Methods and Materials

### Data Source

**Fig. 1:**
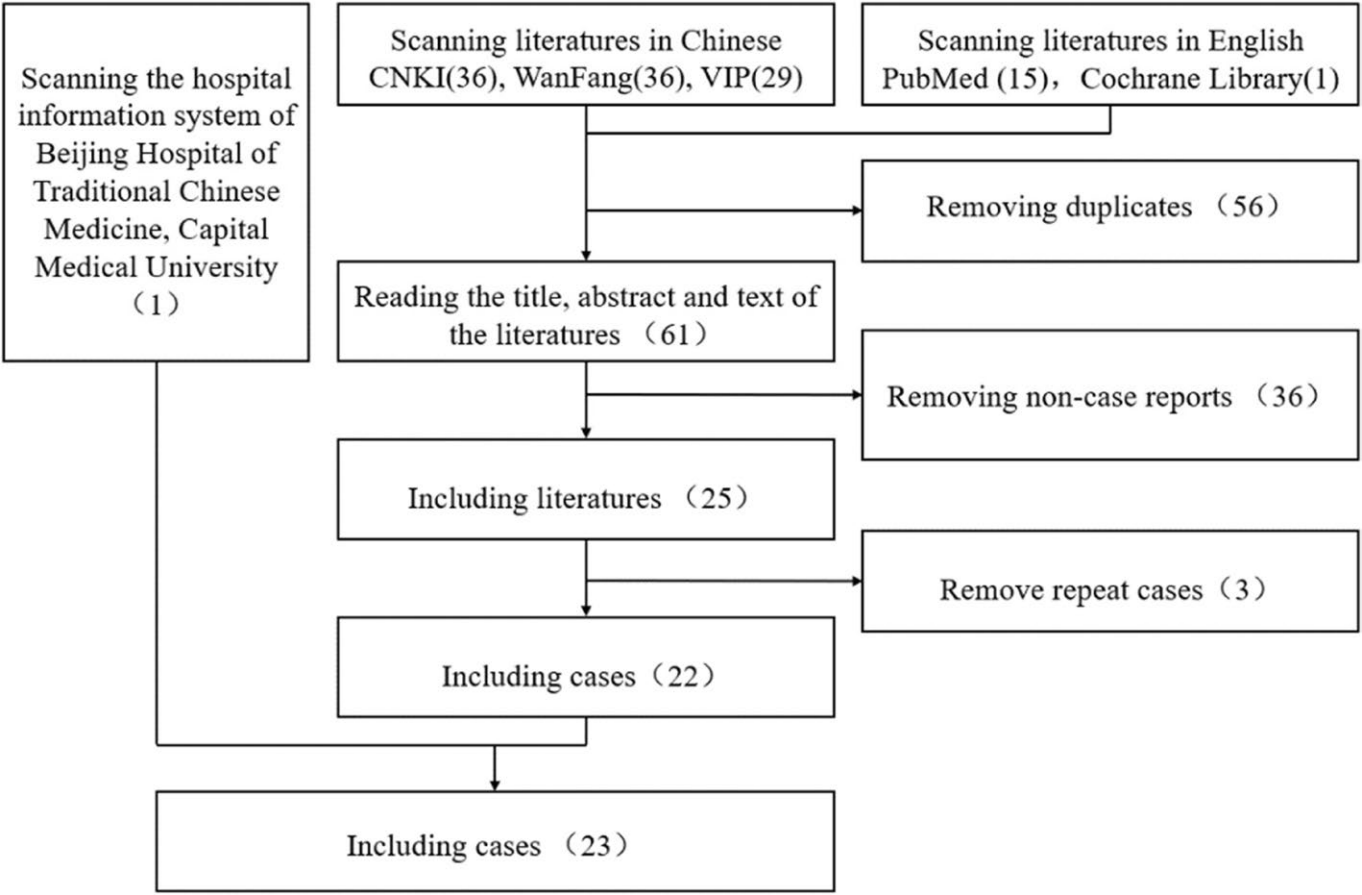
Flow chart of screening cases

The data of this study derived from case reports retrieval and medical record review in hospital information system of Beijing Hospital of Traditional Chinese Medicine. The screening process is depicted in Fig. 1.

The following literature retrieval webs were employed, including: CNKI, Wanfang, VIP, Pubmed and Cochrane Library. Data retrieval was limited to studies in Chinese or in English published before April 1st 2024. The search strategy used terms relating to “sintilimab”, “myocarditis”, “Sindili”, “myocarditis”, “cardiovascular” and “myocardial injury”. A total of 101 Chinese literature and 16 English literature were retrieved. After removing duplicates, 61 studies were retained for further examination. After screening the titles, abstracts and text, 36 studies were excluded because they were not case reports. Consequently, 25 literatures (11 in Chinese and 14 in English) were retained, in which 22 case reports of sintilimab-related autoimmune myocarditis were included with 3 repeated cases removed.

Cases adopting sintilimab in the past three years were searched in the hospital information system of Beijing Hospital of Traditional Chinese Medicine. Included cases fulfilled the *Chinese Expert Consensus on the Surveillance and Management of Immune Checkpoint Inhibitor-Related Myocarditis (2020 version)*clinical diagnostic criteria for ICIs-related autoimmune myocarditis [28]. One case diagnosed as sintilimab-related autoimmune myocarditis with elevated cardiac biomarkers and ECG changes after sintilimab treatment was included.

### Methods

Data extraction was performed using Excel Microsoft software to sort out the data of gender, age, comorbidities, drug dosage, concomitant medications, time of onset, related clinical manifestations and auxiliary examination, treatment, prognosis and other information of the included 23 cases.

Statistical methods: Accordingly, statistical analysis was performed using SPSS Statistics version 27 (IBM Corp., Armonk, NY, USA; account name: Beijing Hospital of TCM, CCUM). Composition ratio, incidence ratio, mortality ratio was presented. Categorical data were compared using the Pearson exact test. Data of coronary heart disease (CHD) and AMI were from *China Cardiovascular Health and Disease Report 2023* [30].

## Result

### Distributions Age and Gender

As listed in Table 1, among the 23 cases of sintilimab-related autoimmune myocarditis, 16 cases (69.57%) were male and seven cases (30.43%) were female. The cases were all aged between 33 and 85 years old, and most of them were elders aging 55 to 85. There was no significant difference between sintilimab-related autoimmune myocarditis and AMI (Table 1).

**Table 1:** The distributions of gender and age of cases with sintilimab-related autoimmune myocarditis comparing with acute myocardial infarction

	Sintilimab-related autoimmune myocarditis (*n* = 23)	Acute myocardial infarction	*P*
Gender			
Male	16 (69.57%)	70.7%	0.520
Female	7 (30.43%)	29.3%	
Age			
< 55 years	7 (30.43%)	23.4%	0.905
55 ~ 85 years	16 (69.57%)	71.5%	

### Comorbidities, Drug Dosage and Concomitant Medications

**Fig. 2:**
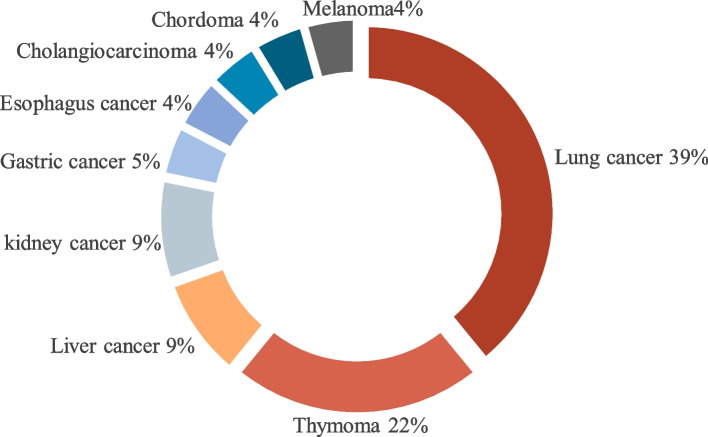
Primary diseases of cases with sintilimab-related autoimmune myocarditis

**Fig. 3:**
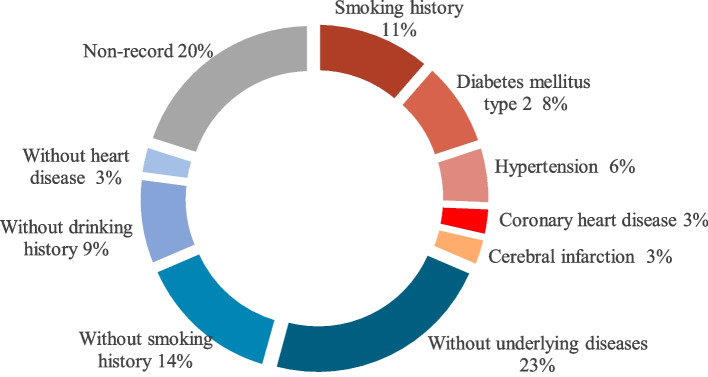
Underlying diseases and cardiovascular risk factors of cases with sintilimab-related autoimmune myocarditis

Among the included 23 cases, all the primary diseases were tumors. The types of tumors included lung cancer (nine cases), thymoma (five cases), liver cancer (two cases) and kidney cancer (two cases) (Fig. 2). Some cases complicated by cardiovascular or cerebrovascular diseases (1 case of coronary heart disease, 1 case of old cerebral infarction) or related with risk factors such as smoking history (four cases), type 2 diabetes (three cases), and hypertension (two cases) (Fig. 3). The doses of sintilimab were all 200 mg, and 14 cases were treated with combination antineoplastic therapy. According to reports, sintilimab was identified as the main cause inducing myocarditis in 14 cases (Tables 2 and 3).

**Table 2:** Comorbid conditions of sintilimab-related autoimmune myocarditis comparing with acute myocardial infarction

	Sintilimab-related autoimmune myocarditis (*n* = 23)	Coronary heart disease	*P*
Type 2 diabetes	3 (13.04%)	26.30%	0.146
Hypertension	2 (8.696%)	60.90%	< 0.0001

**Table 3:** Comorbidities of cases with sintilimab-related autoimmune myocarditis

Comorbidities	Case
Carboplatin	5
Paclitaxel	5
Anlotinib	2
Gemcitabine	2
Oteracil potassium	2
Epirubicin	1
IBI310	1
Axitinib	1
Oxaliplatin	1
Cyclophosphamide	1
Lenvatinib Mesilate	1
Nedaplatin	1
Alimta	1

### Onset Time

As listed in Table 3, most of the sintilimab-related autoimmune myocarditis occurred between the completion of the first treatment cycle and the initiation of the second, especially in 14 days after the end of the first dose. Notably, onset time varies greatly from five days after the first dose of treatment to the end of the fifth dose (Table 4).

**Table 4:** Onset time of sintilimab-related autoimmune myocarditis

Onset time		case(%)
End of the first dose	1 ~ 6 days	1(7.14%)
	7 ~ 13 days	3(21.43%)
	14 days to the beginning of second dose	10(71.43%)
End of the second dose		5(21.74%)
End of the third dose		2(8,70%)
End of the fourth dose		1(4.35%)
End of the fifth dose		1(4.35%)

### Related Clinical Manifestations and Auxiliary Examination

As listed in Table 5, the common clinical manifestations of sintilimab-related autoimmune myocarditis were non-specific symptoms such as chest tightness and shortness of breath (65.22%) and palpitation (30.43%). 47.83% of the patients experienced muscle weakness, ptosis, or dysarthria due to myasthenia, and 17.39% of the patients experienced muscle soreness due to myositis (Table 4). For almost all cases, Cardiac Troponin (Tn), Creatine Kinase (CK), Creatine Kinase isoenzyme MB (CKMB) and Myoglobin (Mb) were significantly elevated in clinical presentation, among which CK and CKMB were elevated in different proportions with CK-MB/CK ratio decreasing. Some cases showed elevated Aspartate Transaminase (AST) and Lactate Dehydrogenase (LDH). A few cases showed the decrease in Left Ventricular Ejection Fraction (LVEF) under transthoracic echocardiography. ECG changes can be observed in the majority of the cases, manifesting as various types of arrhythmias (sinus tachycardia, frequent premature beats, atrioventricular block, bundle branch block, ventricular rhythm, etc.), low voltage in limb leads, QT interval prolongation, ST segment elevation or depression, and T wave deformed. Coronary Angiography (CAG) or coronary Computed Tomography Angiography (CTA) was performed for 9 patients to rule out coronary heart diseases. Cardiovascular Magnetic Resonance (CMR) wad performed in 3 patients to confirm myocarditis, and CMR showed old myocardial injury in 1 patient after treatment improvement (Table 6).

**Table 5:** Manifestations of cases with sintilimab-related autoimmune myocarditis

Manifestations	case	proportion
Chest tightness and shortness of breath	15	65.22%
Muscle weakness, ptosis, dysarthria, etc	11	47.83%
Palpitation	7	30.43%
Muscle soreness	4	17.39%
Cough	2	8.70%
Dizziness	2	8.70%
Abdominal pain	1	4.35%
Fever	1	4.35%
Gross hematuria	1	4.35%
Cognitive decline	1	4.35%
Loss of appetite and weight loss	1	4.35%
Asymptomatic	2	8.70%

**Table 6:** Auxiliary examination of cases with sintilimab-related autoimmune myocarditis

Nomber	TNT (ng/mL)	TNI (ng/mL)	hsTNI (ng/mL)	CK (ng/mL)	CKMB (ng/mL)	MB (ng/mL)	LDH (ng/mL)	ALT (ng/mL)	AST (ng/mL)	BNP (ng/L)	NT-proBNP (ng/L)	LVEF%	CAG /CTA		CMR			Eltromyogram		ECG		ECG changes	Refere-nce
Peipei Rong 2021 [31]	-	-	44.228	-	137.3	> 1000	-	-	-	-	124	-	-		-			-		Sinus tachycardia without any ST-segment changes		-	[31]
Bo Xu 2021 [32]	-	1.68	-	1010	46.3	> 2000		58.9	71.3	-	0.894	-	-		-			-		-		-	[31]
Ling Wu 2022 [33]	-	-	740.4	3864	216.7	-	768.3I	152	281.9	-	-	63	-		-			-		Sinus tachycardia with CRBBB		-	[33]
Fenfen Xu 2022 [34]	-	4.22	-	3425	100	> 2000	584	-	-	-	-	-	-		-			-		Sinus tachycardia with ventricular premature with CRBBB		Severe atrioventricu-lar block	[34]
Huiping Zhou 2022 [35]	-	0.1	-	-	-	54	431.2	-	-	11,914	-	-	-		-			-		Sinus tachycardia		-	[35]
Min Chen 2023 [36]	-	0,1	-	1704	-	-	-	-	-	-	-	-	-		-			-		New atrial premature beat without any ST-segment changes		-	[36]
Ningfu Li 2023 [37]	-	-	0.322	-	117	1975	-	-	-	-	53.29	-	CAT(-)		-			-		Sinus tachycardia with CRBBB		FEPB, NSVT	[38]
Bin Liu 2023 [39]	-	-	0.83	1179	77	-	1182	-	149	-	480.7	-	CAG (-)		-			-		Sinus rhythm with ST segment depression in leads V1-5 with prolongation of QTc interval		-	[39]
Shuangyan Zhang 2023 [40]	0.08	-	-	7830	146	> 2000	937	148	350	57	-	62	-		-			-		Sinus rhythm with T wave low-flat		-	[40]
Yu Zhang 2023 [41]	-	-	109	1821	33.7	313.7	396	51	107	9.13	-	62	CAG (-)		Myocardial edema in ISV and inferior wall			Peripheral		Sinus rhythm with pre-excitaion syndrome type A		-	[41]
Qian Xing 2020 [42]	0.916	-	-	11,919	223.1	> 3000	1210	-	-	117.4	-	-	-		-			-		CRBBB		-	[42, 43]
Huanhuan Bi 2021 [44]	-	2.2	-	2500	140	-	-	-	-	-	-	-	CAG (-)		-			-		Ventricular rhythm with CRBBB with atrioventricular block II°with ST segment depression in multiple leads		-	[44, 45]
Yukai Chen 2021 [46]	1.5	-	-	706	140.7	-	-	168	403	-	581.8	61	-	PET-MR: demonstrated increased FDG metabolism at the base of the left ventricular wall and increased T2WI signals at the base of the ventricular septum					-	Sinus rhythm with ST segment depression in leads I, II, aVF, V4-6, T wave changes inleads I and aVL		-	[46]
Zixuan Yang 2021 [47]	1.566	-	-	25,692	-	-	-	-	-	-	1339	61	-		-			-		Sinus rhythm with new CLBBB		-	[47]
Beibei Yin 2022 [48]	-	2.35	-	1658	124.5	965.4	1616	309.1	154.9	-	-	65	-		CMR 3 months later: old myocardial injuries			-		Atrial rhythm		-	[49]
Shiwei Liu 2022 [49]	-	-	1423.7	7603	53.37	-	805	97	308	-	-	67	-		-			-		Sinus tachycardia with low voltage of the QRS complex in the limb leads	CRBBB, prolongation of QTc interval with changed ST segment		[49]
Yi, Tang 2022 [50]	-	4.1	-	-	-	-	-	-	-	-	1050	45	CAG (-)		-			-		BVT	Advanced atrioventricular block		[50]
Yunling Lin 2022 [51]	-	9.4	-	922	109	-	-	-	-	-	8290	35	CAG (-)		-			-		Sinus rhythm with ST segment elevating in leads V5-9		-	[51]
Siming Zheng 2023 [52]	-	2230	-	4977	526	-	-	-	-	-	8290	64			-			-		Sinus rhythm without any ST-segment changes		-	[52]
Wang, Chen 2023 [53]	-	0.363	-	22,536	748.9	-	1156	-	-	-	-	71	CTA: localized noncalcified plaque in the middle of the left anterior descending artery and mild stenosis of the lumen			-	Amplitude of wave 5 caused by low-frequency re-frequency electrical stimulation on bilateral facial nerves attenuated 5% −10%			Sinus rhythm with prolongation of QTc interval		VT storms, VF	[53]
Xin Liu 2023 [54]	-	-	5574	-	96.7	-	-	-	-	-	111.9	68	CAG (-)		-			-		Sinus rhythm with segment elevating in multiple leads		VT, VF	[54]
Yue Hu 2023 [55]	0.303	-	-	-	-	-	-	-	-	-	-	68	CTA: no evidence of ACS		CMR(-)			-		Sinus rhythm without any ST segment changes		-	[55]
BHTCM 2023	-	5.7	-	2513.8	92.6	278.5	946.6	190.8	349.3	48.27	-	58	-		-			-		Accelerated junctional escape rhythm with ST-segment elevating in multiple leads with NSVT		VT, VE	-

*TNT* Cardiac Troponin T, *TNI* Cardiac Troponin I, *hsTNI* Hypersensitive Troponin I, *CK* Creatine Kinase, *CKMB* Creatine Kinase isoenzyme MB, *MB* Myoglobin, *LDH* Lactate Dehydrogenase, *ALT* Aspartate Transaminase, *AST* Aspartate Transaminase, *BNP* Brain Natriuretic Peptide, *LVEF* Left Ventricular Ejection Fraction, *CAG* Coronary Angiography, *CTA* Computed Coronary Tomography Angiography, *CMR* Cardiovascular Magnetic Resonance, *ECG* Electrocardiogram, *CRBBB* Complete Right Bundle Branch, *FEPB* Frequent Ventricular Premature Beat, *ISV* Interventricular Septum, *CLBBB* complete left bundle branch, *VT* Ventricular Tachycardia, *BVT* Bidirectional Ventricular Tachycardia, *NSVT* Nonsustained Ventricular Tachycardia, *VF* Ventricular Fibrillation, *ACS* Acute Coronary Syndrome, *VE* Ventricular Escape

### Treatment and Prognosis

As listed in Table 6, all included 23 cases quit sintilimab treatment after the occurrence if adverse reactions. And methylprednisolone therapy was given by injection or oral administration in 1 to 14 days after symptom occured. The injection dose ranged from 60 mg/d to 1000 mg/d, and the oral dose was 40 mg/d. Immunoglobulin injection therapy was given to nine cases with 20–30 mg/ day; temporary pacemaker was implanted in four cases; plasma exchange therapy was performed for two cases. Cardiac death occurred in five cases with malignant arrhythmia and peritoneal infection occurred in one case, with a mortality rate of 26.09% (Table 7). Among 17 cases of improvement, four cases were reported still survive in two months after discharged, and one case reported tumor progressed in two months after discharged.

**Table 7:** Treatment and prognosis of cases with sintilimab-related autoautoimmune myocarditis

Nomber	Diagnosis	Systems involved	initiation time	Steroids	Immunoglobulin	Other drugs	Other treatments	Prognosis	Reference
Peipei Rong 2021 [31]	ICIs-related autoautoimmune myocarditis	Circulatory system	-	Methylprednisolone for injection (500 mg/d, gradually decreasing the dose)	-	-	-	Improvement	[31]
Bo Xu 2021 [32]	ICIs-related autoimmune myocarditis Immune myositis, myasthenia Immune liver injury	Circulatory system Moter system Digestive system	8	Methylprednisolone for injection(80 mg/d, gradually decreasing the dose)	-	Magnesium isoglycyrrhizinate, Adenosine cyclophosphate	-	Improvement	[31]
Ling Wu 2022 [33]	ICIs-related autoimmune myocarditis	Circulatory system	-	Methylprednisolone for injection(1 g/d, gradually decreasing the dose)	Immunoglobulin (20 mg/d)	-		Improvement	[33]
Fenfen Xu 2022 [34]	Immune myositis ICIs-related autoimmune myocarditis	Circulatory system Moter system	3	Methylprednisolone for injection(240 mg/d for 5 days, gradually decreasing the dose)	-	-	Temporary pacemaker	Improvement	[34]
Huiping Zhou 2022 [35]	ICIs-related autoimmune myocarditis	Circulatory system	8	Methylprednisolone for injection(160 mg/d, gradually decreasing the dose)	-	-	-	Improvement	[35]
Min Chen 2023 [36]	ICIs-related autoimmune myocarditis myasthenia	Circulatory system Moter system	6	Methylprednisolone for injection(60 mg/d, gradually decreasing the dose)	-	-	-	Improvement	[36]
Ningfu Li 2023 [37]	ICIs-related autoimmune myocarditis	Circulatory system	3	Methylprednisolone for injection(500 mg/d for 4 days, gradually decreasing the dose)	Immunoglobulin (20 mg/d)	Trimetazidine, Coenzyme Q10, Sacubitril valsartan sodium	-	Cardiac death	[37, 38]
Bin Liu 2023 [39]	ICIs-related autoimmune myocarditis	Circulatory system	3	Methylprednisolone for injection(120 mg/d, gradually decreasing the dose)	-	-	-	Improvement	[39]
Shuangyan Zhang 2023 [40]	ICIs-related autoimmune myocarditis Immune myositis	Circulatory system + Moter system	5	Methylprednisolone for injection (500 mg/d, gradually decreasing the dose)				Improvement	[40]
Yu Zhang 2023 [41]	ICIs-related autoimmune myocarditis Immune myositis, myasthenia	Circulatory system Moter system	1	Methylprednisolone for oral(40 mg/d, gradually decreasing the dose)	-	-	-	Improvement	[41]
Qian Xing 2020 [42]	ICIs-related autoimmune myocarditis Immune myositis	Circulatory system Moter system	-	Methylprednisolone for injection(500 mg/d, gradually decreasing the dose)	Immunoglobulin (25 g/d)	Pyridostigmine Bromide120g Qid	Temporary pacemaker, Plasmapheresis	Improvement	[42, 43]
Huanhuan Bi 2021 [44]	ICIs-related autoimmune myocarditis	Circulatory system	-	Methylprednisolone for injection (80 mg/d for 1 days、60 mg/d for 3 days、40 mg/d for 5 days) Methylprednisolone for oral (16 mg/d for 15 days、8 mg/d for 45 days)	-	-	-	Improvement	[44, 45]
Yukai Chen 2021 [46]	ICIs-related autoimmune myocarditis Immune myositis、myasthenia Immune liver injury	Circulatory system Moter system Digestive system	5	Methylprednisolone for injection(480 mg/d for 5 days) Methylprednisolone for oral (40 mg/d for 28 days)	-	-	-	Dead of abdominal infection	[46]
Zixuan Yang 2021 [47]	ICIs-related autoimmune myocarditis	Circulatory system Moter system	6	Methylprednisolone for injection(2 mg/kg/d for 5 days, gradually decreasing the dose)	Immunoglobulin (20 g/d for 5 days)	Pyridostigmine Bromide 180 mg/d	-	Improvement	[47]
Beibei Yin 2022 [48]	ICIs-related autoimmune myocarditis Immune myositis、myasthenia	Circulatory system Moter system	-	Methylprednisolone for injection(500 mg/d for 5 days, gradually decreasing the dose)	Immunoglobulin (0.4 g/kg/d for 5 days)	Tacrolimus(3 mg/d)	-	Improvement	[49]
Shiwei Liu 2022 [49]	ICIs-related autoimmune myocarditis	Circulatory system	1	Methylprednisolone for injection(100 mg/d for 3 days, gradually decreasing the dose)	Immunoglobulin (30 mg for 1 days)	Coenzyme Q10	-	Cardiac death	[49]
Yi, Tang 2022 [50]	ICIs-related autoimmune myocarditis	Circulatory system	-	Methylprednisolone for injection(200 mg/d, gradually decreasing the dose)	Immunoglobulin	-	Temporary pacemaker,	Cardiac death	[50]
Yunling Lin 2022 [51]	ICIs-related autoimmune myocarditis	Circulatory system	-	Methylprednisolone for injection(2 mg/kg/d for 7 days, gradually decreasing the dose)	Immunoglobulin (0.4 g/kg/d for 7 days)	-	-	Improvement	[51]
Siming Zheng 2023 [52]	ICIs-related autoimmune myocarditis	Circulatory system	3	Methylprednisolone for injection(15 mg/d) after CAG ruling out ACS (120 mg/d, gradually decreasing the dose)	-	Aspirin Atorvastatin	-	Improvement	[52]
Wang, Chen 2023 [53]	ICIs-related autoimmune myocarditis myasthenia	Circulatory system Moter system	3	Methylprednisolone for injection(120 mg/d, gradually decreasing the dose)	-	-	Cardioversion Plasmapheresis	Cardiac death	[53]
Xin Liu 2023 [54]	ICIs-related autoimmune myocarditis	Circulatory system	14	Methylprednisolone for injection(120 mg/d, gradually decreasing the dose)	Immunoglobulin (20 g for 7 days)	-	IABP	Improvement	[54]
Yue Hu 2023 [55]	ICIs-related autoimmune myocarditis	Circulatory system	-	Methylprednisolone for injection(1 mg/kg/d for 3 days, gradually decreasing the dose)	-	-	-	Improvement	[55]
BHTCM 2023	ICIs-related autoimmune myocarditis	Circulatory system Moter system	4	Methylprednisolone for injection(80 mg/d for 3 days)	-	-	Temporary pacemaker	Cardiac death	-

*ICIs* Immune Checkpoint inhibitors, *CAG* Coronary Angiography, *ACS* Acute Coronary Syndrome, *IABP* Intra-aortic balloon pump

## Discussion

Characterized by sudden onset and rapid progression, Sintilimab-related autoimmune myocarditis is similar with AMI in the affected popularity and clinical manifestations, which brings significant difficulties to differentiate the two. However, the therapeutic principles of them are quite different. Therefore, differential diagnosis of the tow rapidly and accurately is of great value. Sintilimab-related autoimmune myocarditis has a preponderance of elderly males, which may be related to the characteristics of the primary disease treated by sintilimab (such as non-small cell lung cancer, etc.) [56]. AMI frequently occurs at midlife and beyond, and the mortality rate of men is higher than that of women [56, 57]. Obviously, there is an overlap between sintilimab-related autoimmune myocarditis and AMI in susceptible popularity. The common clinical manifestations of autoimmune myocarditis are mostly non-specific symptoms similar to AMI, such as chest tightness and shortness of breath, palpitations, etc. Some patients even only present elevated cardiac biomarkers such as Tn without obvious symptoms. Elevated cardiac biomarkers only indicates the myocardial damage and is not satisfactory in the specific diagnosis of autoimmune myocarditis [58, 59]. The history of sintilimab therapy is the key to the identification of sintilimab-related autoimmune myocarditis. However, the scattered onset time brings more difficulty to the diagnosis and differential diagnosis. Moreover, there may be interaction between sintilimab-related autoimmune myocarditis and AMI. Studies have shown that people with AMI risk factors such as heart disease and diabetes may increase the risk of myocarditis, while hypercoagulability induced by tumors may promote coronary thrombosis [18], and ICIs may also accelerate atherosclerosis and plaque rupture [60]. These dilemmas make the differential diagnosis of the two even more difficult.

Since the incidence of AMI is much higher than sintilimab-related autoimmune myocarditis [15, 56], the latter is easily misdiagnosed as AMI in clinical practice. Clarify the medical history of sintilimab therapy is essential in the diagnosis. As shown in Table 4, all patients with sintilimab therapy, whether in early (< 90 days after ICI therapy initiation) or in late (≥ 90 days after ICI therapy initiation) therapy period, should be alert to the possibility of autoimmune myocarditis when cardiac related symptoms such as chest tightness appearing. Myocarditis occurs in 17.39% cases after the third dose of treatment, and late adverse events is mainly revealed to be heart failure [56, 61]. Past history is helpful to identify autoimmune myocarditis. Comparing to patients with autoimmune myocarditis, patients with AMI are more likely to have cardiovascular risk factors such as hypertension, diabetes, and smoking. Moreover, in addition to cardiovascular adverse reactions, the adverse reactions of sintilimab often involve multiple systems such as exercise and digestion [62]. As a result, a note of caution on autoimmune myocarditis should be added when patients show evidence other than cardiovascular system.

Exclusionary diagnosis is one of the important way to distinguish sintilimab-related autoimmune myocarditis from AMI, due to its poor specificity in symptoms, signs, laboratory tests and electrocardiogram [28]. CAG is the gold standard for diagnosing AMI [26], but CAG exclusion in the diagnosis of autoimmune myocarditis has disadvantages in clinical practice. First of all, patients may not withstand the invasive CAG examination due to their poor condition as a result of the long-term consuming of tumor. Secondly, patients with myocarditis may delay their seeking medical attention because of the non-specific symptoms, which may easily result in missing the emergency CAG owing to the misdiagnosis of AMI beyond the time window for emergency intervention. CTA is a non-invasive examination with high negative predictive value for coronary artery disease [63, 64]. However, coronary disease is easily overestimated in elderly patients by CTA, because of the significant affection coronary artery calcification make the assessment of coronary artery stenosis degree, which is very common in elderly patients [65]. Unfortunately, tumor is common in elderly population, which increases the risk of misdiagnosing autoimmune myocarditis as AMI due to false positive CTA result, especial without the basement image.

Myocardial biopsy is the gold standard for the diagnosis of autoimmune myocarditis [66–68], but not widely used in clinical practice especially in acute and critical myocarditis, owning to its invasiveness and risk of cardiac perforation. CMR is the gold standard for non-invasive diagnosis of myocarditis caused by traditional etiology [66, 69]. But studies have shown that its sensitivity in the diagnosis of ICI related myocarditis may decrease, especially in the early stage of the disease, where false negatives are prone to occur [70].

Some other features of autoimmune myocarditis are also helpful in clinical diagnosis. Cardiac biomarkers have been proved to be valuable in the early diagnosis of autoimmune myocarditis [58, 71, 72]. Tn often presents a continuous increasing before steroids therapy [58], which was significantly different from the of elevate-peak-fall like variation of Tn in AMI [73]. CK-MB/CK ratio usually decrease, and the multiple of CK-MB exceeding the normal upper limit is usually lower than that of CK, which is significantly different from the proportional rise of the two of AMI. And this may be related to the release of other isoenzymes of CK due to muscle injury caused by immune myositis, which is often been seen with sintilimab-related autoimmune myocarditis [74, 75]. Moreover, the elevating of Tn and CK can be a predicter of poor prognosis. Studies have shown that patients with autoimmune myocarditis who present a Tn of ≥ 1.5 ng/ml was associated with a fourfold increased risk of MACE [18] Hypersensitive troponin may have higher potential value in the diagnosis and prognosis prediction of autoimmune myocarditis [76, 77]. In addition, studies have shown that the elevating of AST, Glutamic Pyruvic Transaminase (ALT), LDH and other non-cardiac biomarkers can also play a certain reference value in auxiliary diagnosis and prognosis judgment [58]. Brain Natriuretic Peptide (BNP) levels are of limited clinical value, which depends on whether the myocardial damage leads to heart failure. The electrocardiogram of patients with sintilimab-related autoimmune myocarditis often shows various types of arrhythmias, with or without ST-T changes, among which atrioventricular block and bundle branch block have certain value in diagnostic specificity [18]. New severe conduction block and ventricular rhythm may suggest poor prognosis, which presents in all four cases of cardiogenic death included in the study. It should be noted that, the measurement of the LVEF would not provide any utility in diagnosis or prognosis prediction, only 2 of the 23 patients reported significant decrease in LVEF. Studies have shown that LVEF were normal in 50% patients with autoimmune myocarditis, and in 38% patients with cardiac events [18].

Sintilimab is often used in combination with antineoplastic drugs such as chemotherapeutic drugs, anti-angiogenic drugs, and other ICIs, and multi-drug combination increases the risk of autoimmune myocarditis. Studies have shown that morbidity and mortality of autoimmune myocarditis were higher with combination sintilimab plus other ICIs [78, 79]. The combination of sintilimab plus anti-angiogenic drugs such as antilotinib may also aggravate myocardial damage [39]. In addition, some antineoplastic drugs may cause other cardiac adverse reactions. Anthracyclines may cause dose-dependent heart failure and irreversible myocardial damage due to its cardiotoxic [80, 81]. Hypertension is a common adverse reaction of anti-angiogenic drugs, with a clinical incidence of up to 56.2%. It has been reported that AMI and aortic dissection may occurred after hypertension onsets [82–84]. Therefore, for patients with combination therapy of sintilimab and other antineoplastic drugs, it is necessary to be especially vigilant with the autoimmune myocarditis caused by overlap syndrome, and attention should be paid in identifying the cardiac adverse reactions caused by different antineoplastic drugs.

Glucocorticoids are recommended as the first choice for ICIS-related myocarditis [28]. *American Society of Clinical Oncology Clinical Practice Guideline*points out that, it is essential to use glucocorticoids early and adequately, which can improve the prognosis of autoimmune myocarditis [17]. However, among the 23 cases included in this study, the initiation time of the 5 cases whose outcomes reported cardiogenic death was 3–4 days, while the outcomes of the other 4 cases whose initiation time was more than 8 days were reported improved. This may be related to the different severity in myocarditis. Patients with insidious onset and mild involvement often delay their treatment due to inconspicuous symptoms, resulting in delayed initiation medication. On the contrary, although patients with sudden onset and severe involvement seek medical treatment in time due to distinct symptoms, they still die of the rapid progression of the disease after intensive treatment. Therefore, rapid and accurate diagnosis of autoimmune myocarditis, especially severe autoimmune myocarditis, and timely treatment are of great significance in clinical practice.

The mortality rate of sintilimab related autoimmune myocarditis is relatively high, with previous reports suggesting which ranges between 39.7% and 51% [85, 86]. And the main causes of death are malignant arrhythmias, including advanced atrioventricular bloc, ventricular escape, ventricular tachycardia, ventricular fibrillation. The mechanism of malignant arrhythmia is still unclear, which may be related to the damage of the cardiac conduction system caused by the autoimmune reaction induced by sintilimab [50]. In addition, the cachexia caused by the tumor may lead to electrolyte disturbance, which plays an important role in promoting malignant arrhythmia. Among the five patients with cardiogenic death, three patients developed heart failure after malignant arrhythmia, manifesting as increased BNP or NT-proBNP, and one patient showed a mild decrease in EF, which was consistent with previous studies [18]. This suggests that one of the causes of death in patients with autoimmune myocarditis may be heart failure with preserved EF or heart failure with mid-range EF. It is worth noting that infection may be a risk factor for death in patients with autoimmune myocarditis. Among the dead cases, one was caused by abdominal infection, and the other two cases were accompanied by respiratory infection. Therefore, clinical attention should be paid to adverse reactions such as infection after the application of glucocorticoids.

## Limitation

First, most of the cases in this study were case reports, which leads to lacking of control group and pretty convincing statistical analysis. Besides, some data is not fully reported in literature. Meanwhile, the sample size is small (only 23) increasing the unreliable generalizability of the conclusions. In summary, larger sample size, more standardized retrospective studies are needed for further in-depth research.

## Conclusion

In summary, sintilimab-related autoimmune myocarditis is prone to be misdiagnosis in clinical practice especially with AMI due to its lacking of specific manifestation. Patients with chest pain who have a history of sintilimab therapy or presenting other systemic manifestations should be alert to the possibility of autoimmune myocarditis. If condition permitting, CAG or coronary CTA should be performed as soon as possible to rule out AMI, CMR or even myocardial biopsy can be performed directly to confirm the diagnosis. Meanwhile, a note of caution should be added to elevating cardiac biomarkers and changes in electrocardiogram for the assistant of diagnosis, and a comprehensive judgment can made by referring to AST, ALT, and LDH.

## Data Availability

No datasets were generated or analysed during the current study.

## Abbreviations

ALT: Glutamic Pyruvic Transaminase
AMI: Acute Myocardial Infarction
AST: Aspartate Transaminase
BNP: Brain Natriuretic Peptide
CAG: Coronary Angiography
CK: Creatine Kinase
CKMB: Creatine Kinase isoenzyme MB
CMR: Cardiovascular Magnetic Resonance
CTA: Computed Tomography Angiography
ECG: Electrocardiogram
ICIs: Immune Checkpoint inhibitors
LDH: Lactate Dehydrogenase
LVEF: Left Ventricular Ejection Fraction
Mb: Myoglobin
PD-1: Programmed cell death receptor-1
PDL-1: Programmed cell death receptor ligand-1
Tn: Cardiac Troponin
